# Hypertrophie bilatérale du canal du Wharton révélatrice d'un Syndrome de Gougerot Sjögren

**DOI:** 10.11604/pamj.2015.22.170.8103

**Published:** 2015-10-21

**Authors:** Naziha Khammassi, Imen Souissi

**Affiliations:** 1 Service de Médecine Interne, Faculté de médecine de Tunis, Hôpital Razi 2010, La Manouba, Tunisie

**Keywords:** Syndrome de Gougerot Sjögren, hypertrophie des glandes sous maxillaires, hypertrophie des canaux de Wharton, Sjögren syndrome, hypertrophy submandibular glands, hypertrophy of Wharton ducts

## Image en medicine

Le canal de Wharton draine les secrétions de la glande sous-maxillaire. Les étiologies de la dilatation de ce canal sont nombreuses: mégacanaux salivaires idiopathiques, lithiase salivaire, dilatation canalaire résultant d'une sténose tumorale ou traumatique et calcinose salivaire. Cette dernière est due à la présence de multiples concrétions parenchymateuses bilatérales dans un groupe de glandes. Il existerait une relation entre calcinose et Syndrome de Gougerot Sjögren (SGS). Patiente de 55 ans sans antécédents pathologiques notables était hospitalisée pour exploration d'une hypertrophie du canal de Wharton et une sécheresse buccale et oculaire évoluant depuis un an. L'examen somatique notait une hypertrophie des glandes sous maxillaires, une hypertrophie des canaux de Wharton surtout à droite sans calcul palpable sur leur trajet et l'orifice de Wharton était libre. Le reste de l'examen était sans particularités. La scintigraphie salivaire montrait une perfusion et une captation faible des glandes parotides et sous maxillaires, cet aspect est en faveur d'un syndrome sec. L’échographie cervicale était sans anomalies. Le bilan immunologique (anticorps antinucléaires, anti SSA, anti SSB, facteur rhumatoïde) était positif. Le «Break up time» (BUT) était altéré à 3’‘ en faveur d'une xérophtalmie. La biopsie des glandes salivaires accessoires objectivait une sialadénite stade III de Chisholm confirmant le diagnostic du SGS et la patiente a été mise sous traitement symptomatique.

**Figure 1:**
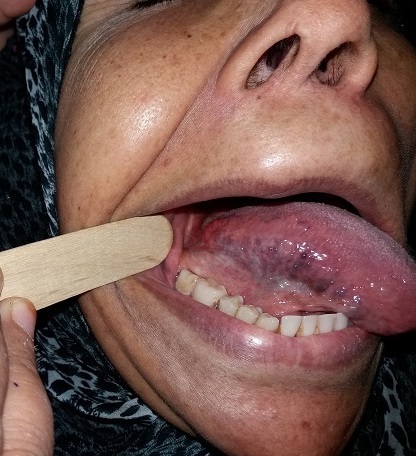
Hypertrophie du canal de Wharton

